# Editorial: Bridging the gap between basic neurosciences and clinical neuroimmunology

**DOI:** 10.3389/fnins.2023.1285678

**Published:** 2023-10-26

**Authors:** Gianmarco Abbadessa, Alessandro Bombaci, Alberto Gajofatto, Simona Bonavita

**Affiliations:** ^1^Department of Advanced Medical and Surgical Sciences, University of Campania “Luigi Vanvitelli”, Naples, Italy; ^2^Department of Brain Sciences, Faculty of Medicine, Imperial College London, London, United Kingdom; ^3^“Rita Levi Montalcini” Department of Neuroscience, University of Torino, Turin, Italy; ^4^Department of Neurosciences, Biomedicine and Movement Sciences, University of Verona, Verona, Italy

**Keywords:** neuroscience, biomarkers, basic research, translational research, neurology, neuroimmunology

The field of neurology continues to advance rapidly, driven by a growing body of scientific knowledge. Bridging the gap between basic science and clinical neurology is crucial for advancing our understanding of neurological disorders and translating research findings into practical applications for patient care. Basic science forms the foundation of our knowledge about the fundamental mechanisms underlying the brain and nervous system functioning. It investigates the principles of neuroscience studying cellular and molecular processes through animal models, and other experimental approaches. On the other hand, clinical neurology focuses on the diagnosis, treatment, and management of neurological disorders in human subjects.

Despite their interdependence, there is a divide between basic science and clinical practice. This gap may arise due to differences in methodology, language, priorities, and resources between researchers working at the bench and clinicians working at the bedside. Bridging this gap requires collaboration and communication between scientists and clinicians to integrate their expertise and perspectives.

To address this gap and contribute to bridging the divide between basic science and clinical neurology, this Research Topic aimed to collect research articles reporting efforts in translational research in neuroscience. This editorial sheds light on certain aspects, elucidating barriers and potential benefits.

One major barrier between basic research findings and clinical practice is the significant costs and lack of standardization associated with new biomarkers that are not routinely applied. The economic constraints and complexity of testing for these biomarkers hinder their widespread adoption in healthcare settings, limiting their potential impact on patient care and treatment outcomes. For instance, previous research has demonstrated the predictive value of several serum or cerebrospinal fluid (CSF) indicators in multiple sclerosis (MS) relapse recurrence. However, the testing process for most indicators is expensive and complex, highlighting the need to identify economically advantageous markers. In this direction, recent investigations have also revealed that the neutrophil-to-lymphocyte ratio (NLR), platelet-to-lymphocyte ratio (PLR), monocyte-to-lymphocyte ratio (MLR), monocyte × NLR (systemic inflammation response index, SIRI), and platelet × NLR (systemic immune inflammation index, SII) serve as inflammation markers and can predict the prognosis of various tumors and also various inflammatory disorders (Guzel et al., 2016; Gong et al., 2021). The role of these markers has already been partially described in patients with MS. In a recent study, it has been demonstrated that four of these inflammatory indices (NLR, PLR, MLR, SII) are positively associated with Gd-enhancing lesions on T1 brain Magnetic Resonance Imaging (MRI) (Gokce et al., 2023). A previous study showed the changes of these two indices in relation to clinical activity, radiological findings, EDSS progression, and brain atrophy, indicating that elevated NLR and MLR may be used as independent markers for the severity of MS-related neurological disability and MRI outcomes (Hemond et al., 2019). The article by Fang et al. focused on the role of peripheral neuroinflammation in Neuromyelitis Optica Spectrum Disorders (NMOSD). The study identifies several markers, including neutrophil count, NLR, PLR, SII, that are elevated in NMOSD patients compared to those with MS and healthy individuals. The research also highlights the potential of PLR and MLR as markers for disease activity and recurrence in NMOSD. The identification of specific blood markers, such as neutrophil count, NLR, PLR, and SII, in NMOSD patients may have important implications for clinical practice. These markers not only help distinguish NMOSD from other conditions but also provide insights into disease severity, activity, and recurrence. However, while these biomarkers offer promise due to their affordability and accessibility, particularly in less specialized clinical settings, their specificity is under scrutiny. Using them for diagnosis, prognosis, and treatment warrants caution due to potential confounding factors and overlapping expressions. Indeed, the complex interactions of these markers within various inflammatory conditions require a deeper understanding of their mechanisms and potential cross-reactivity with other disorders.

Another interesting area of research that has grown in the last decades involves exploring the potential implications of cellular fetal microchimerism in wound healing, autoimmunity, cancer, and possibly cardiovascular disease. This emerging field presents a novel perspective on maternal and transgenerational health and provides exciting opportunities for developing new disease biomarkers and precision medicine with targeted prophylaxis against long-term maternal disease. Bianchi et al. (*Microchimerism in multiple sclerosis: the association between sex of offspring and MRI features in women with multiple sclerosis*) explored the presence of fetal microchimeric cells (fMCs) in women with MS. The study indicates an association between the sex of offspring, an indirect marker of fMCs, and MRI features in MS patients. The authors hypothesized that their findings could be related to the fMCs that can migrate to the mother during pregnancy and accumulate in different brain areas. These fMCs may modulate MS neuropathological processes. By identifying an indirect marker of fMCs through the sex of offspring, the study provides hypotheses about possible mechanisms modulating neuropathological processes in MS. This finding may bridge the gap by highlighting the potential long-term effects of fetal cells on maternal health and suggesting that considering pregnancy history may be relevant in understanding and managing MS. However, it's important to acknowledge that these findings are based on indirect markers and correlations. To establish a more definitive understanding of the impact of fMCs in MS and other diseases, additional research studies are essential.

Looking at basic science findings, over the last few decades, studies on murine models have brought to light a multitude of potential biomarkers. While these models offer valuable opportunities to delve deeper into pathological molecular mechanisms, it is crucial to acknowledge the considerable gap that exists between these research findings and their translation into clinical practice. In the article by Yu et al. the authors explored the role of the proinflammatory cytokine Interleukin-17A (IL-17A) in heart failure (HF). The study demonstrates that elevated expression of IL-17A in the brain, specifically in the hypothalamic paraventricular nucleus (PVN), contributes to neuroinflammation and cardiac dysfunction in HF. Researchers found that suppressing the IL-17A/IL-17RA axis in the brain significantly reduced mRNA expression of the measured inflammatory cytokines and chemokines in the PVN in HF rats. They suggest that interventions targeting this pathway could hold promise in the treatment of HF. By elucidating the mechanism by which IL-17A acts within the brain, specifically in the hypothalamic PVN, to promote sympathetic outflow, the study provides a potential target for therapeutic interventions. This finding may bridge the gap by identifying a specific molecular pathway within the central nervous system that can be targeted for intervention in clinical settings. However, it is essential to acknowledge that despite these promising findings, there is still a significant distance to cover before translating these discoveries into clinical practice.

Further examples of research offering opportunities to bridge this gap come from studies delving into the molecular pathways of immune homeostasis.

Procaccini et al. (2021) conducted a study focusing on the role of metabolic and transcriptional regulation in Treg (regulatory T cell) function. Their research significantly advances our understanding of Treg cell biology and offers translational insights, particularly for autoimmune diseases like relapsing-remitting multiple sclerosis (RRMS). A key finding is the identification of SLC7A11 (xCT) as a critical regulator in Treg cells. SLC7A11 induction in Treg cells depends on the mTOR pathway and NRF2 activation. This induction plays a central role in Treg cell proliferation by managing reactive oxygen species (ROS) and facilitating metabolic reprogramming, including enhanced glycolysis and glucose utilization. This result highlight SLC7A11 relevance in enabling Treg cells to respond to environmental cues and proliferate under pseudo-starvation conditions. The study's observations regarding Treg cell dysfunction in RRMS patients have direct implications for autoimmune disease research and treatment. Treg cells from RRMS patients showed impaired proliferation and reduced antioxidant responses, aligning with previous studies findings. Crucially, the study suggests a promising therapeutic avenue by identifying SLC7A11 as a key regulator of Treg cell function. This insight can guide the development of interventions aimed at modulating Treg cell activity. Notably, the study demonstrated that dimethyl fumarate (DMF), an NRF2-targeting drug, effectively restored Treg cell function in RRMS patients, offering a potential treatment strategy for autoimmune diseases. This research contributes to the concept of precision medicine in autoimmune disease management. Understanding the molecular differences between healthy and disease affected Treg cells enables more precise treatment approaches. Researchers can explore how these differences manifest in individual patients, potentially leading to personalized treatment strategies tailored to each patient's Treg cell molecular profile. In conclusion, the identification of SLC7A11 as a central regulator of Treg cell function opens doors for future studies and targeted therapies in immune-related diseases. Another example comes from the study by Vigo et al. (2019) that investigated how IFNβ influences the therapeutic potential of mesenchymal stem cells (MSCs), potentially as an add-on therapy. IFNβ not only boosted the immunomodulatory capabilities of MSCs but also triggered the expression of key mediators like Slpi and Hgf, involved in immune and regenerative functions. At the molecular level, IFNβ rapidly activated STAT1 and STAT3, known transcription factors for Slpi and Hgf. Simultaneously, IFNβ had dynamic effects on mTOR activity, initially suppressing it but later inducing it, accompanied by increased glycolysis in MSCs. When MSCs shifted their metabolism toward glycolysis, they gained enhanced control over T-cell proliferation. These findings suggest that IFNβ-induced metabolic changes may enhance MSCs' immunomodulatory functions, indicating a potential combined therapy approach in conditions like MS, where IFNβ could augment MSCs' immunoregulatory and regenerative activities.

However, it's crucial to note that these effects have primarily been observed in *in vitro* settings. The transition to *in vivo* environments introduces greater complexity. While these effects have been observed in murine models, it is important to recognize that the disease in animal models may not fully reflect the complexity of human disease. Additionally, the intricate nature of human diseases often cannot be fully addressed by single-molecule or cascade-focused approaches alone. In the near future, omic sciences will likely be integrated into basic science, potentially addressing this unmet need and facilitating the translation of these findings into effective clinical interventions. Navigating these challenges in the context of the multifaceted nature of human diseases will remain essential.

Finally, in our Research Topic of studies aimed at providing biomarkers for clinical practice, it is crucial to recognize the value of neurophysiological basic studies like the one conducted by Sharmin et al.. While the focus of the investigation may differ from the immediate goal of biomarker identification, it exemplifies the broader perspective that neurophysiological research can offer. Sharmin et al. study on nerve conduction velocity (NCV) and stretch presents intriguing possibilities for understanding nerve function, which could potentially be translated into clinical practice. By exploring the novel conduction mechanism based on nodal resistance, Sharmin et al.'s work provides valuable insights into neurophysiology that may contribute to the development of innovative biomarkers or therapeutic approaches in the field of clinical neurology.

In conclusion our Research Topic provides some insights into the complex relationship between basic science and clinical neurology. It highlights the importance of understanding the role of inflammatory mediators both in cardiac and neurological diseases, the role of fetal microchimeric cells, and nerve conduction mechanisms. Further, the collected article highlights the numerous barriers in translating basic science findings into clinical practice but also offer the opportunity to shed light on the potential benefits of bridging this gap. Indeed, it could open new avenues for potential therapeutic interventions and personalized approaches in the field of neurology. Continuous research in this direction will undoubtedly lead to improved patient outcomes and a deeper understanding of neurological diseases.

## Author contributions

GA: Conceptualization, Writing—original draft. AB: Writing—original draft. AG: Writing—review & editing. SB: Conceptualization, Supervision, Writing—review & editing.
